# Associations of Maternal Iron Intake and Hemoglobin in Pregnancy with Offspring Vascular Phenotypes and Adiposity at Age 10: Findings from the Avon Longitudinal Study of Parents and Children

**DOI:** 10.1371/journal.pone.0084684

**Published:** 2014-01-06

**Authors:** Nisreen A. Alwan, Janet E. Cade, Darren C. Greenwood, John Deanfield, Debbie A. Lawlor

**Affiliations:** 1 Nutritional Epidemiology Group, School of Food Science and Nutrition, University of Leeds, Leeds, United Kingdom; 2 Division of Epidemiology and Biostatistics, Leeds Institute for Health, Genetics and Therapeutics, University of Leeds, Leeds, United Kingdom; 3 Vascular Physiology Unit, Institute of Cardiovascular Science, University College London, London, United Kingdom; 4 Medical Research Council Integrative Epidemiology Unit & School of Social and Community Medicine, University of Bristol, Bristol, United Kingdom; National Institute of Agronomic Research, France

## Abstract

**Background:**

Iron deficiency is common during pregnancy. Experimental animal studies suggest that it increases cardiovascular risk in the offspring.

**Objective:**

To examine the relationship between maternal pregnancy dietary and supplement iron intake and hemoglobin, with offspring’s arterial stiffness (measured by carotid-radial pulse wave velocity), endothelial function (measured by brachial artery flow mediated dilatation), blood pressure, and adiposity (measured by body mass index), test for mediation by cord ferritin, birth weight, gestational age, and child dietary iron intake, and for effect modification by maternal vitamin C intake and offspring sex.

**Design:**

Prospective data from 2958 mothers and children pairs at 10 years of age enrolled in an English birth cohort, the Avon Longitudinal Study for Parents and Children (ALSPAC), was analysed.

**Results:**

2639 (89.2%) mothers reported dietary iron intake in pregnancy below the UK reference nutrient intake of 14.8 mg/day. 1328 (44.9%) reported taking iron supplements, and 129 (4.4%) were anemic by 18 weeks gestation. No associations were observed apart from maternal iron intake from supplements with offspring systolic blood pressure (−0.8 mmHg, 99% CI −1.7 to 0, P = 0.01 in the sample with all relevant data observed, and −0.7 mmHg, 99% CI −1.3 to 0, P = 0.008 in the sample with missing data imputed).

**Conclusion:**

There was no evidence of association between maternal pregnancy dietary iron intake, or maternal hemoglobin concentration (which is less likely to be biased by subjective reporting) with offspring outcomes. There was a modest inverse association between maternal iron supplement intake during pregnancy with offspring systolic blood pressure at 10 years.

## Introduction

Iron is an essential micronutrient important in carrying oxygen, and to the catalytic activity of a variety of enzymes. In the fetus, it is used to synthesize hemoglobin, and is integral to brain development [1]. Iron deficiency is the most common nutrient deficiency in the world [2], with 25–40% of pregnant women in Western societies estimated to have iron deficiency [3]. Iron deficiency anemia in pregnancy has been linked to low birthweight, preterm delivery and perinatal iron deficiency anemia [4], [5], [6], [7], [8].

These short-term adverse effects may reflect an impact of iron deficiency on placental structure and function, which may have long-term effects via fetal organ development [9]. Evidence from animal models suggests that maternal iron deficiency during pregnancy can result in the development of obesity and hypertension in the offspring [10], [11], [12], [13], but evidence in humans remains inconclusive. In a previous analysis of data from the same birth cohort we analysed, the Avon Longitudinal Study of Parents and Children (ALSPAC) cohort, Brion et al reported an association between maternal anemia and offspring blood pressure (BP) at 7 years in women who did not take iron supplements during pregnancy [14]. Belfort et al, with a sample size of 1167 US pregnant women, did not find an association between first and second trimester maternal anemia with offspring BP at 3 years. However, offspring BP was positively associated with first trimester iron intake, in contrast to animal studies findings [10], [11], [12], [13], while no relationship was observed for second trimester iron intake [15]. Other studies examining the association between maternal anemia in pregnancy and offspring childhood BP reported conflicting findings ranging from positive to negative, and including null associations [16], [17], [18], [19].

Maternal iron status in pregnancy is strongly associated with umbilical cord ferritin [20], [21], [22]. Thus, cord ferritin could mediate any intrauterine effect of maternal iron status on later offspring cardiovascular outcomes. Animal studies suggest that the inverse association of maternal iron status with later offspring cardiovascular outcomes differs by offspring gender, being stronger in males [11], [23]. Furthermore, since vitamin C is a key enhancer of iron absorption [24], [25], the relationship between maternal iron intake and perinatal or longer term outcomes may be stronger with adequate intake of vitamin C [26].

We aim to examine the relationship between indicators of maternal iron status in pregnancy and indicators of child’s circulatory health. In this study, we extended the work done by Brion et al using ALSPAC data [14], aiming to examine the associations between maternal iron intake and hemoglobin concentrations during pregnancy with offspring‘s BP at a later age (10 years) than the previous analysis, plus including offspring adiposity as assessed by body mass index, and other offspring vascular indicators; endothelial function as assessed by brachial artery flow mediated dilatation, and arterial stiffness as assessed by carotid-radial pulse wave velocity. We examined whether any observed associations were mediated by cord blood ferritin levels, gestational age, offspring’s birthweight, and dietary iron intake and explored whether offspring sex and maternal vitamin C intake moderate any associations.

## Subjects and Methods

ALSPAC is a longitudinal, population-based birth cohort study which recruited pregnant women (n = 14541 pregnancies) from the South West of England between 1990 and 1992 and has followed the parents and offspring since then. The cohort is described in detail elsewhere [27], [28]. Please note that the study website contains details of all the available data through a fully searchable data dictionary **(** www.bris.ac.uk/alspac/researchers/data-access/data-dictionary). Ethical approval was obtained from the ALSPAC Law and Ethics Committee and the local research ethics committees, and procedures were in accordance with the Helsinki Declaration of 1975 as revised in 1983. Participants provided their written informed consent to participate in this study.

**Figure 1:**
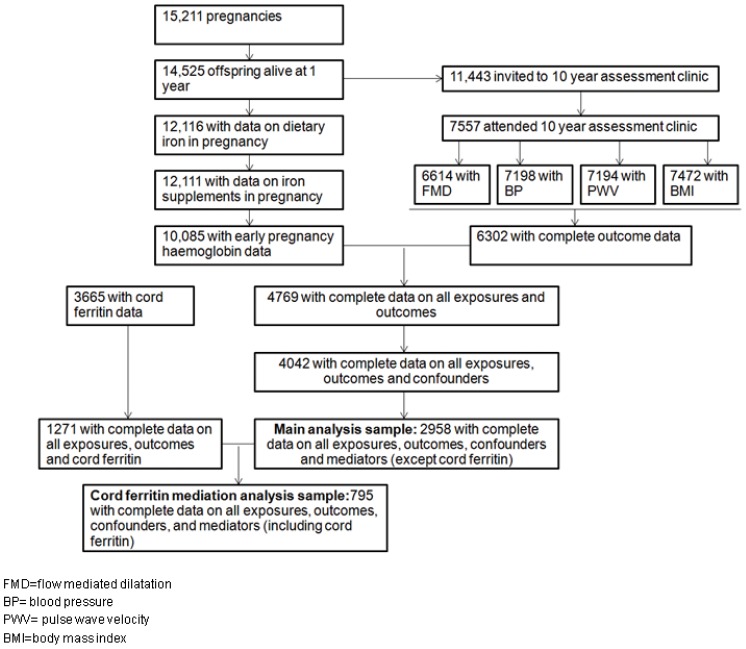
Participants flow chart.

Figure 1 shows participants flow-chart for the complete case analysis performed for this study.

### Exposure Assessment

Iron intake from food was assessed using a food-frequency questionnaire (FFQ) sent to mothers at 32 weeks gestation covering all the main foods consumed in Britain. Mothers were asked how often they were currently consuming each of 43 food groups [29]. Intakes for a range of nutrients, including iron, were derived using nutrient information on standard-sized portions based on the 5^th^ edition of McCance and Widdowson’s Composition of Food tables [30]. The FFQ was used to calculate an approximate daily nutrient intake for each woman by multiplying the weekly frequency of consumption of a food by the nutrient content. The nutrient values obtained were then divided by seven to convert this to a daily intake including energy, protein, total fat, saturates, monounsaturates and polyunsaturates, total sugar, non-milk extrinsic sugar, dietary fibre, nine vitamins and five minerals [29].

Maternal iron supplement use was obtained from questionnaires sent at 18 weeks gestation (relating to anytime during pregnancy before the questionnaire date), and 32 weeks gestation (relating to 3 months of pregnancy between the first and second questionnaire). Mothers were asked whether they had taken iron supplements, vitamins, or any other supplements. In a separate question, women were also asked to list all pills, medicines, and ointments they used, with a reminder to include iron tablets, vitamins, herbal medicines, etc. Responses at 18 and 32 weeks regarding iron supplements were combined to generate a binary variable (yes/no) for ‘iron supplement used anytime during pregnancy up to 32 weeks gestation’.

Maternal hemoglobin concentrations were extracted from antenatal medical records of study participants as it was measured routinely in all pregnancy women. An ‘Early pregnancy hemoglobin’ variable was derived, defined as the first measurement of hemoglobin before 18 weeks. A mother was classified as having ‘early pregnancy anemia’ if her hemoglobin measurement was less than 11 g/dl, the threshold used to define pregnancy anemia according to World Health Organization guidelines [31].

### Outcome Assessment

BP, pulse wave velocity, flow mediated dilatation and body mass index were measured in child clinics at ages 10–11 years by six trained research technicians/fieldworkers over a two year study period [32]. Carotid-radial pulse wave velocity was measured transcutaneously using a high-fidelity micromanometer (SPC-301, Millar Instruments, Houston, TX, USA). Ultrasound images of the right brachial artery were used to measure flow mediated dilatation percentage. The coefficients of variation between technicians for flow mediated dilatation and pulse wave velocity were 10.5% and 4.6% respectively at the beginning of the study, and reached 7.7% and 4.1% at the end of the study. Detailed description of the measurement methods is available elsewhere [32]. Flow mediated dilatation reflects endothelium-dependent vasodilator function, which is considered prognostic of cardiovascular risk [33], [34]. Pulse wave velocity measures the time taken for the systolic pressure wave to travel a known distance, and is increasingly considered a convenient, reliable and integrated index of vascular pathology over the lifecourse [35]. Weight to the nearest 0.1 kg was measured in light clothing and without shoes using SECA scales. Height to the nearest 0.1 cm was measured using a Leicester height meter. From these, body mass index was calculated (weight in kg/height in metres^2^).

### Covariables

Current age of the child was recorded in months at the time of the assessment clinic. Child sex was recorded at birth from the obstetric records. We considered the following covariables to be potential confounders (associated with both exposures and outcomes); maternal age, pre-pregnancy body mass index, educational level (as a marker of socioeconomic status), smoking in pregnancy, parity, and maternal total energy intake as assessed by food frequency questionnaire at 32 weeks.

Highest maternal educational qualification was self-reported at 32 weeks gestation, and was categorized as university degree, A-level or equivalent (A-level is Advanced-level and indicates a qualification usually taken around 18 years of age by individuals who have remained in school beyond the legal minimum age at which they can leave (16 years) and are likely to go on to higher or further education or train for a semi-skilled job), and less than A-level. Maternal smoking was self-reported at the 18 and 32 weeks gestation questionnaire. A variable was generated for any smoking during pregnancy reported at either or both of these time points.

On the basis of it being plausible that maternal iron status would affect them and that they could plausibly causally affect the offspring’s cardiovascular profile [36], [37], we considered the following potential mediators of the association; cord ferritin, gestational age, offspring birthweight and offspring dietary iron intake. Gestational age at delivery and birthweight were obtained from the obstetric records. Ferritin was measured in cord heparin plasma at the ALSPAC laboratory using the DELFIA time resolved fluoroimmunoassay system. Ferritin assays were duplicated where possible and a coefficient of variation of approximately 4% was obtained. Offspring dietary iron intake was assessed by a food frequency questionnaire administered at 3, 4, 7 and 9 years of age [38]. We used the mean iron intake of these 4 assessments. Maternal vitamin C intake (which we considered a potential effect modifier of maternal iron status on offspring outcomes) was calculated from the food frequency questionnaire as described above for iron intake. A binary variable for dietary vitamin C intake was created using the UK Reference Nutrient Intake cut-off of 50 mg/day and used to test for interaction [39]. Baby’s sex (also a potential effect modifier) was obtained from the birth records.

### Statistical Analysis

Analysis was performed using Stata version 11 (StataCorp LP, College Station, TX, USA). Characteristics of women with iron intake above or equal to the reference nutrient intake were compared to those with intake below the reference nutrient intake using two-sample t-test for continuous variables and chi-squared test for categorical variables.

Linear regression was used to examine the association of maternal dietary iron intake (assessed as a continuous variable per 10 mg/day and also as a binary variable<versus ≥14.8 mg/day), whether or not the mother took iron supplements in pregnancy and early pregnancy hemoglobin (assessed as a continuous 1 g/dL and as a binary variable<versus ≥11 g/dL) with offspring pulse wave velocity, flow mediated dilatation, BP and body mass index all measured at mean age 10 years. Initially, univariable (no adjustment for covariables) analyses were undertake followed by multivariable models that adjusted first for potential confounding factors (maternal age, pre-pregnancy body mass index, educational level, smoking in pregnancy, parity, and maternal total energy intake for analyses involving dietary iron intake as the exposure), then for potential mediation by birthweight, gestational age and offspring postnatal dietary iron intake. Finally, in the subgroup with data on cord ferritin levels, its role as a mediator was examined.

The possibilities that offspring sex or maternal adequate intake of vitamin C (≤ versus >50 mg/day) modified the association of maternal exposures with offspring outcomes were assessed by examining stratified (by sex and maternal adequate vitamin C intake) analyses and by including an interaction term in the confounder adjusted models.

A statistical significance level of 1%, with 99% confidence intervals, was used in the regression models to reduce the risk of type I error due to multiple statistical testing.

### Sensitivity Analyses

We undertook sensitivity analysis aimed at exploring whether missing data might have led to biased estimates. To do this, we used multivariable multiple imputation in Stata as described by Royston [40] to impute missing values for variables included in the main analysis models for any ALSPAC participant in the 12116 sample with dietary iron intake data. Twenty imputation datasets were generated in which missing variables values were imputed by chained equations including exposures, outcomes and covariables as used in the main confounder-adjusted models. The sensitivity analysis results are obtained by averaging over the results from each of the 20 datasets using Rubin’s rules [40].

We also carried out another sensitivity analysis, adding maternal hemoglobin to the models exploring the associations of maternal iron supplement intake with childhood outcomes. This was to account for potential reverse causality (the reason for taking the iron supplements is the mother’s awareness of being anaemic).

## Results

The main analysis sample was 2958 with complete data on all exposures, outcomes, confounders and mediators (except for cord ferritin). A description of the study sample characteristics, compared with the total ALSPAC sample with dietary data is shown in table 1. Mean maternal pregnancy iron intake was 10.7 mg/day (standard deviation = 3.2). The mean age of the child at the time of the focus clinic was 119 months (9.9 years, standard deviation = 0.3). Table 2 summarized participants’ characteristics by maternal iron intake. Women with dietary iron intake equal to or more than the UK Reference Nutrient Intake for iron were more likely to be older, vegetarian, with higher educational qualification, report higher total energy and vitamin C intake, have lower pre-pregnancy body mass index, and were less likely to smoke during pregnancy.

**Table 1:** Study sample characteristics (complete cases for exposures, outcomes, confounders and mediators n = 2958) and ALSPAC sample characteristics (with dietary iron intake data n = 12116).

	Complete case sample	ALSPAC sample (with dietary data)
**Dietary iron intake** # **(mg/day) (m, sd** * **)**	10.7 (3.2)	10.4 (3.4)
**Dietary iron intake <UK RNI** ## **(%, 95% CI** ** **)**	89.2 (88.0, 90.3)	90.3 (89.7, 90.8)
**Dietary iron intake <UK LRNI** ### **(%, 95% CI)**	18.0 (16.7, 19.5)	25.0 (24.2, 25.8)
**Dietary vitamin C intake** ## **(mg/day) (m, sd)**	85.9 (34.5)	80.1 (35.3)
**Age of mother (yrs) (m, sd)**	29.5 (4.2)	28.5 (4.8)
**Pre-pregnancy BMI (kg/m^2^) (m, sd)**	22.8 (3.5)	22.9 (3.6)
**Total energy intake (kj) (m, sd)**	7,483 (1,908)	7,415 (2,081)
**Smoking in pregnancy (%, 95% CI** ** **)**	15.4 (14.1, 16.7)	25.2 (24.4, 26.0)
**Caucasian (%, 95% CI)**	98.8 (98.4, 99.2)	97.5 (97.2, 97.8)
**University degree (%, 95% CI)**	18.6 (17.2, 20.0)	13.8 (13.1, 14.4)
**Vegetarian (%, 95% CI)**	6.1 (5.3, 7.0)	5.2 (4.8, 5.6)
**Primigravida (%, 95% CI)**	48.8 (47.0, 50.6)	44.8 (43.9, 45.7)
**Early pregnancy maternal anemia (<11 g/dL) (%, 95% CI)**	4.4 (3.7, 5.1)	5.1 (4.6, 5.5)
**Report taking iron supplements Before 32 wks gestation (%, 95% CI)**	44.9 (43.1, 46.7)	47.5 (46.6, 48.4)
**Child gender (male) (%, 95% CI)**	50.8 (49.0, 52.6)	51.5 (50.6, 52.4)
**Birthweight (g) (m, sd)**	3,448 (499)	3,411 (544)
**Gestational age (wks) (m, sd)**	39.6 (1.6)	39.5 (1.8)

^#^ Food frequency questionnaire at 32 weeks gestation.

^##^ UK Reference Nutrient Intake (14.8 mg/day).

^###^ UK Lower Reference Nutrient Intake (8 mg/day).

Mean, standard deviation.

95% confidence intervals.

**Table 2:** Sample characteristics by dietary iron intake as assessed by food frequency questionnaire at 32 weeks gestation (n = 2958 for all, except where cord ferritin data is used: n = 795).

	Dietary iron intake		
	≥14.8 mg/day# (n = 319)	<14.8 mg/day (n = 2,639)	P value*
**Dietary iron intake** ## **(mg/day) (m, sd** ** **)**	16.9 (1.9 )	10.1 (2.5)	–
**Dietary vitamin C intake** ## **(mg/day) (m, sd)**	119.3 (35.6)	81.8 (32.1)	<0.001
**Age of mother (yrs) (m, sd)**	30.0 (4.2)	29.5 (4.2)	0.02
**Pre-pregnancy BMI (kg/m^2^) (m, sd)**	21.7 (2.5)	22.9 (3.6)	<0.001
**Total energy intake (kj) (m, sd)**	10,100 (1,921)	7,167 (1,645)	<0.001
**Smoking in pregnancy (%, 95% CI** *** **)**	10.7 (7.5, 14.6)	15.0 (14.6, 14.4)	0.001
**Caucasian (%, 95% CI)**	98.1 (96.0, 99.3)	99.0 (98.4, 99.3)	0.2
**University degree (%, 95% CI)**	27.0 (22.2, 32.2)	17.6 (16.2, 19.1)	<0.001
**Vegetarian (%, 95% CI)**	11.9 (8.3, 16.6)	5.5 (4.6, 6.4)	<0.001
**Primigravida (%, 95% CI)**	48.0 (42.4, 53.6)	51.0 (47.0, 50.9)	0.8
**Early pregnancy maternal anemia (<11 g/dL) (%, 95% CI)**	6.6 (4.1, 9.9)	4.1 (3.4, 4.9)	0.04
**Report taking iron supplements Before 32 wks gestation (%, 95% CI)**	48.3 (42.7, 53.9)	44.5 (42.6, 46.4)	0.2
**Cord ferritin (µg/L) (m,sd)**	151.9 (104.8) (n = −83)	165.4 (123.6) (n = 712)	0.3
**Child systolic blood pressure (mmHg) (m, sd)**	103.9 (8.7)	103.6 (8.8)	0.5
**Child diastolic blood pressure (mmHg) (m, sd)**	60.3 (7.3)	59.5 (7.8)	0.08
**Child pulse wave velocity (m/s) (m, sd)**	7.7 (1.3)	7.5 (1.2)	0.1
**Child flow mediated dilatation (%) (m, sd)**	8.3 (3.5)	8.0 (3.4)	0.1
**Child body mass index (kg/m^2^) (m, sd)**	17.9 (2.7)	18.1 (3.0)	0.2
**Child gender (male) (%, 95% CI)**	55.2 (49.5, 60.7)	50.3 (48.3, 52.2)	0.1
**Birthweight (g) (m, sd)**	3,439 (486)	3,450 (500)	0.7
**Gestational age (wks) (m, sd)**	39.5 (1.6)	39.6 (1.6)	0.3

^#^ Reference nutrient intake (RNI) for iron for women aged 19–50 years in the UK.

^##^ Food frequency questionnaire at 32 weeks gestation.

P-value using two-sample t-test for continuous variables, chi-squared test for categorical variables.

Mean, standard deviation.

95% confidence intervals.

In unadjusted analyses, maternal dietary iron intake was associated with offspring body mass index, and maternal pregnancy supplement intake was associated with offspring systolic BP. With adjustment for confounding characteristics, there were no associations between the primary exposures and outcomes of interest apart from the inverse association between maternal iron supplement intake and offspring systolic BP which remained largely unchanged, with marginal statistical significance at the 1% significance level (0.8 mmHg lower with reporting taking iron supplements, 99% CI 0 to 1.7, P = 0.01). This association was not markedly affected by adjustment for mediators (birthweight, gestational age, and offspring dietary iron intake), but was attenuated to the null in the smaller sub-sample with further adjustment for cord ferritin as a fourth mediator (table 3).

**Table 3:** Linear regression estimates for associations between maternal iron intake and hemoglobin in pregnancy with offspring vascular indicators and body mass index (n = 2958 for all, except where cord ferritin data is used: n = 795).

		Offspring pulse wavevelocity (m/s)				Offspring flow mediateddilatation (%)								Offspring systolic bloodpressure (mmHg)								Offspring diastolic bloodpressure (mmHg)					Offspring body massindex (kg/m^2^)				
		B	99% CI®	P		B	99% CI®			P				B		99% CI®			P			B	99% CI®		P		B		99% CI®		P
**Maternal pregnancy dietary iron intake (continuous) (per 10 mg/d)**																															
Unadjusted		0.2	0, 0.4	0.02		0.2	−0.4, 0.6			0.5			−0.6		−1.9, 0.7			0.3				0.4	−0.8, 1.5	0.4		−0.8		−1.3, −0.4		<0.001	
Model 1#		0.1	−0.2, 0.4	0.5		0.4	−0.3, 1.2			0.1			−0.3		−2.2, 1.7			0.7				0.7	−1.1, 2.4	0.3		0.1		−.0.5, 0.7		0.7	
Model 2*		0.1	−0.2, 0.4	0.3		0.5	−0.3, 1.2			0.1			−0.1		−2.1, 2.0			0.9				1.0	−0.8, 2.8	0.2		0.1		−0.6, 0.7		0.8	
Model 3**		0.5	−0.1, 1.1	0.04		0.3	−1.2, 1.9			0.6			0.7		−3.5, 4.9			0.7				1.2	−2.4, 4.8	0.4		0.2		−1.1, 1.5		0.7	
**Maternal iron intake <14.8 mg/d**																															
Unadjusted		−0.1	−0.3, 0.1	0.1	−0.3		−0.8, 0.2			0.1		−0.3				−1.7, 1.0			0.5		−0.8		−2.0, 0.4		0.08	0.2			−0.2, 0.7		0.2
Model 1#		0	−0.3, −0.2	0.7	−0.5		−1.1, 0.1			0.05		−0.7				−2.3, 0.8			0.3		−0.9		−2.3, 0.4		0.08	−0.4			−0.9, 0.1		0.03
Model 2*		0	−0.3, 0.2	0.7	−0.5		−1.1, 0.1			0.04		−0.8				−2.3, 0.8			0.2		−1.0		−2.4, 0.3		0.05	−0.4			−0.9, 0.1		0.03
Model 3**		−0.1	−0.6, 0.3	0.5	0.1		−1.0, 1.2			0.8		−0.8				−3.9, 2.2			0.5		−1.2		−3.9, 1.5		0.3	−0.5			−1.5, 0.4		0.2
**Maternal pregnancy iron supplement use**																															
Unadjusted		−0.1	−0.1, 0.2	0.07	0.2			−0.1, 0.5		0.1		−1.0				−1.8, −0.1			0.003		−0.4		−1.1, 0.3		0.2	−0.1			−0.4, 0.2		0.3
Model 1#		0.1	−0.1, 0.2	0.2	0.2			−0.1, 0.6		0.07		−0.8				−1.7, 0			0.01		−0.3		−1.1, 0.4		0.3	0			−0.2, 0.3		0.7
Model 2*		0.1	−0.1, 0.2	0.1	0.2			−0.1, 0.6		0.06		−0.8				−1.6, 0.1			0.02		−0.2		−1.0, 0.5		0.5	0			−0.3, 0.3		0.9
Model 3**		0	−0.2, 0.3	0.7	0.1			−0.6, 0.7		0.8		−0.9				−2.6, 0.8			0.2		0.1		−1.4, 1.5		0.9	−0.2			−0.7, 0.3		0.4
**Maternal early pregnancy hemoglobin (g/dL)**																															
Unadjusted		−0.1	−0.1, 0	0.07	−0.1				−0.2, 0.1		0.5			0.4			−0.1, 0.8			0.05		0	−0.4, 0.4		0.8	0.1			0, 0.3		0.06
Model 1#		0	−0.1, 0	0.09	−0.1				−0.3, 0.1		0.3			0.2			−0.3, 0.7			0.2		0	−0.5, 0.4		0.8	0			−0.2, 0.1		0.7
Model 2*		−0.1	−0.1, 0	0.08	−0.1				−0.3, 0.1		0.3			0.2			−0.3, 0.7			0.3		−0.1	−0.5, 0.4		0.7	0			−0.2, 0.2		0.9
Model 3**		−0.1	−0.2, 0.1	0.3	0				−0.4, 0.3		0.8			0.3			−0.6, 1.3			0.4		−0.1	−0.9, 0.7		0.7	0.1			−0.2, 0.4		0.6
**Maternal early pregnancy anemia (<11 g/dL)**																															
Unadjusted	0		−0.3, 0.3	0.8	−0.2				−1.0, 0.6		0.5			−1.5			−3.6, 0.6			0.06		0.4	−2.2, 1.4		0.5	−0.1			−0.8, 0.6		0.6
Model 1#	0		−0.3, 0.3	0.8	−0.2				−1.0, 0.6		0.6			−1.3			−3.3, 0.8			0.1		−0.3	−2.1, 1.5		0.6	0.1			−0.5, 0.8		0.7
Model 2*	0		−0.3, 0.3	0.8	−.0.2				−0.9, 0.6		0.6			−1.2			−3.2, 0.9			0.1		−0.2	−2.0, 1.6		0.7	0			−0.6, 0.7		0.8
Model 3**	0		−0.7, 0.3	0.2	−0.1				−1.4, 1.3		0.9			−1.0			−4.6, 2.6			0.5		−0.3	−3.4, 2.8		0.8	0.1			−1.0, 1.2		0.7

^#^ Adjusting for confounders: maternal age, pre-pregnancy body mass index, smoking in pregnancy, educational qualification (as a proxy for socioeconomic status), parity (and maternal total energy intake in the models with dietary iron intake as exposure).

Adjusting for confounders and three mediators: birth weight, gestational age, offspring dietary iron intake.

Adjusting for confounders and four mediators: birth weight, gestational age, offspring dietary iron intake, cord ferritin.

®confidence intervals.

The main adjusted associations were largely similar when conducted using the multiple imputation databases (table 4), compared to those conducted on complete data presented as primary analyses in this paper (table 3). However, adjusting for cord ferritin as a mediator in the inverse association between maternal iron supplement use and offspring systolic BP in the imputed dataset had less impact than in the complete dataset (table 4).

**Table 4:** Multivariable linear regression for associations between maternal iron intake and hemoglobin in pregnancy with offspring vascular indicators and body mass index using multiple imputation dataset based on the sample with dietary iron intake data (n = 12116).

		Offspring pulse wavevelocity (m/s)				Offspring flow mediateddilatation (%)								Offspring systolic bloodpressure (mmHg)								Offspring diastolic bloodpressure (mmHg)					Offspring body massindex (kg/m^2^)				
		B	99% CI®	P		B	99% CI®			P				B		99% CI®			P			B	99% CI®		P		B		99% CI®		P
**Maternal pregnancy dietary iron intake (continuous) (per 10 mg/d)**																															
Unadjusted		0	−0.1, 0.1	0.3		0.1	−0.3, 0.4			0.6			−1.0		−1.9, −0.1			0.004				−0.5	−1.3, 0.3	0.1		−0.7		−1.1, −0.4		<0.001	
Model 1#		−0.1	−0.2, 0.1	0.5		0.5	−0.1, 1.0			0.03			0.2		−1.9, 1.4			0.7				−0.3	−1.6, 1.0	0.6		0.2		−0.3, 0.7		0.3	
Model 2*		0	−0.4, 0.3	0.5		0.5	−0.1, 1.0			0.02			0		−1.6, 1.6			0.9				0	−1.3, 1.4	0.9		0.1		−0.4, 0.6		0.6	
Model 3**		0	−0.2, 0.1	0.5		−0.2	−0.1, 1.1			0.6			0.1		−1.7,1.6			0.9				0	−1.4, 1.4	0.9		0		−0.4, 0.6		0.6	
**Maternal iron intake <14.8 mg/d**																															
Unadjusted		−0.1	−0.2, 0	0.06	−0.2		−0.6, 0.2			0.1		0.6				−0.4, 1.6			0.1		0		−0.9, 0.9		0.9	0.3			0.1, 0.6		0.002
Model 1#		−0.1	−0.2, 0.1	0.3	−0.4		−0.8, 0.1			0.03		0				−1.3, 1.3			0.9		−0.3		−1.3, 0.7		0.5	−0.2			−0.5, 0.1		0.06
Model 2*		−0.1	−0.2, 0.1	0.2	−0.4		−0.8, 0.1			0.03		−0.1				−1.3, 1.2			0.9		−0.4		−1.4, 0.6		0.3	−0.2			−0.5, 0.1		0.08
Model 3**		−0.1	−0.2, 0.1	0.2	−0.4		−0.8, 0.1			0.02		−0.1				−1.3, 1.2			0.9		−0.4		−1.4, 0.6		0.3	−0.2			−0.5, 0.1		0.08
**Maternal pregnancy iron supplement use**																															
Unadjusted		0.1	0, 0.1	0.2	0.1			−0.1, 0.4		0.2		−0.8				−1.4, −0.2			0.001		−0.3		−0.8, 0.2		0.2	−0.2			−0.4, 0		0.002
Model 1#		0	−0.1, 0.1	0.3	0.1			−0.1, 0.4		0.1		−0.7				−1.3, 0			0.008		−0.2		−0.7, 0.4		0.4	0			−0.2, 0.2		0.9
Model 2*		0	−0.1, 0.1	0.2	0.2			−0.1, 0.4		0.08		−0.6				−1.2, 0			0.01		−0.1		−0.7, 0.4		0.5	−0.1			−0.2, 0.1		0.5
Model 3**		0	0, 0.1	0.2	0.2			−0.1, 0.4		0.08		−0.6				−1.3, 0			0.01		−0.2		−0.7, 0.4		0.4	0			−0.2, 0.1		0.5
**Maternal early pregnancy hemoglobin (g/dL)**																															
Unadjusted		0	−0.1, 0	0.1	0				−0.1, 0.1		0.8			0.3			−0.1, 0.7			0.05		0.1	−0.3, 0.4		0.6	0.1			0, 0.3		0.02
Model 1#		0	−0.1, 0	0.3	0				−0.1, 0.1		0.9			0.2			−0.2, 0.5			0.3		0	−0.3, 0.3		0.8	−0.1			−0.2, 0.1		0.2
Model 2*		0	−0.1, 0	0.3	0				−0.1, 0.1		0.9			0.1			−0.2, 0.5			0.3		0	−0.4, 0.3		0.8	0			−0.2, 0.1		0.3
Model 3**		0	−0.1, 0	0.3	0				−0.1, 0.1		0.9			0.1			−0.2, 0.5			0.3		0	−0.3, 0.3		0.8	−0.1			−0.2, 0.1		0.3
**Maternal early pregnancy anemia (<11 g/dL)**																															
Unadjusted	0		−0.3, 0.2	0.6	0				−0.6, 0.6		0.9			−0.1			−1.5, 1.2			0.8		0	−1.4, 1.2		0.9	0.1			−0.5, 0.6		0.8
Model 1#	−0.1		−0.3, 0.2	0.6	0				−0.6, 0.6		0.9			0.1			−1.2, 1.5			0.8		0.2	−1.2, 1.6		0.8	0.4			−0.2, 0.9		0.07
Model 2*	−0.1		−0.3, 0.2	0.5	0				−0.6, 0.6		0.9			0.1			−1.2, 1.5			0.8		0.1	−1.3, 1.5		0.8	0.4			−0.2, 0.9		0.08
Model 3**	−0.1		−0.3, 0.2	0.5	0				−0.6, 0.6		0.9			0.1			−1.2, 1.5			0.8		0.2	−1.2, 1.6		0.7	0.4			−0.2, 0.9		0.07

^#^ Adjusting for confounders: maternal age, pre-pregnancy body mass index, smoking in pregnancy, educational qualification (as a proxy for socioeconomic status), parity (and maternal total energy intake in the models with dietary iron intake as exposure).

Adjusting for confounders and three mediators: birth weight, gestational age, offspring dietary iron intake.

Adjusting for confounders and four mediators: birth weight, gestational age, offspring dietary iron intake, cord ferritin.

®confidence intervals.

Adjusting for early pregnancy maternal hemoglobin in the association between maternal iron supplement use and offspring systolic BP as sensitivity analysis did not change the magnitude of association (−0.8 mmHg, 99% CI 0.1 to −1.7, P = 0.03 in the main complete dataset, and −0.6 mmHg, 99% CI 0 to −1.3, P = 0.01 in the imputed dataset).

There was also no evidence of effect modification by maternal vitamin C intake or child sex on any of the relationships (table 5). There was also no association between cord ferritin and either maternal dietary iron intake or hemoglobin.

**Table 5:** Multivariable linear regression estimates from stratified analyses for associations between maternal iron intake and hemoglobin in pregnancy with offspring vascular indicators and body mass index with testing for effect modification by maternal vitamin C intake during pregnancy and child sex (n = 2958).

		Offspring pulse wavevelocity (m/s)				Offspring flow mediateddilatation (%)								Offspring systolic bloodpressure (mmHg)								Offspring diastolic bloodpressure (mmHg)						Offspring body massindex (kg/m^2^)				
		B#	99% CI®	P*		B#	99% CI®			P*				B#		99% CI®			P*			B#	99% CI®			P*		B#		99% CI®		P*
**Maternal pregnancy dietary iron intake (continuous) (per 10 mg/d)**																																
In participants with vitamin Cintake >50 mg/day		0	−0.3, 0.3	0.5		0.6	−0.2, 1.5			0.5			−0.3		−2.4, 1.9			0.7				0.8	−1.1, 2.7		0.8		0		−.0.7, 0.6		0.3	
In participants with vitamin Cintake ≤50 mg/day		0.2	−0.7, 1.1			−0.3	−2.7, 2.1						−1.8		−7.3, 3.7							1.2	−4.0, 6.4				1.2		−1.1, 3.5			
Males		0.2	−0.2, 0.6	0.4		0.6	−0.4, 1.6			0.2			0.4		−2.3, 3.1			0.9				0.9	−1.5, 3.2		0.7		0.2		−0.7, 1.0		0.4	
Females		0	−0.4, 0.4			0.5	−0.7, 1.6						−0.9		−3.8, 2.0							0.7	−1.9, 3.3				0.1		−0.9, 1.0			
**Maternal pregnancy iron supplement use**																																
In participants with vitamin Cintake >50 mg/day		0	−0.1, 0.2	0.2	0.3			0, 0.7		0.1		−0.8				−1.7, 0.1			0.9		−0.4		−1.2, 0.5			0.9	0.1			−0.2, 0.3		0.9
In participants with vitamin Cintake ≤50 mg/day		0.2	−0.1, 0.5		−0.3			−1.1, 0.6				−0.8				−2.7, 1.2					−0.1		−2.0, 1.8				0			−0.8, 0.8		
Males		0.1	−0.1, 0.2	0.8	0.2			−0.2, 0.6		0.9		−0.9				−2.1, 0.3			0.7		−0.9		−1.9, 0.1			0.06	−0.1			−0.4, 0.3		0.5
Females		0.1	−0.1, 0.2		0.3			−0.3, 0.7				−0.7				−1.9, 0.5					0.2		−0.8, 1.3				0.1			−0.3, 0.5		
**Maternal early pregnancy anemia (<11 g/dL)**																																
Males	0		−0.4, 0.4	0.9	0.2				−0.9, 1.2		0.3			−1.3			−4.1, 1.4			0.8		0.3		−2.1, 2.6		0.4	0.4			−0.5, 1.2		0.2
Females	−0.1		−0.5, 0.4		−0.5				−1.7, 0.7					−1.0			−4.1, 2.1					−0.9		−3.7, 1.8			−0.2			−1.2, 0.8		

^#^ Adjusting for potential confounding characteristics: maternal age, pre-pregnancy BMI, smoking in pregnancy, educational qualification (as a proxy for socioeconomic status), parity, and maternal total energy intake in the models with dietary iron intake as exposure).

®confidence intervals.

Interaction P value, testing the null hypotheses that associations do not differ by maternal vitamin C level or by sex of participants.

## Discussion

In this study, we have examined associations of maternal pregnancy iron intake and early-pregnancy hemoglobin with several markers of offspring cardiovascular health at 10 years. Of the 25 main associations that we examined only one was observed, that of an inverse association of maternal report of taking a supplement that contained iron before 32 weeks gestation with offspring systolic BP at 10 years of age. However, although the direction of the latter association is consistent with results from previous animal studies [10], [11], [12], [13], its magnitude was modest (1 mmHg lower offspring systolic BP on average in children whose mothers reported taking supplements compared to those who did not). To take some account of multiple testing, we have used a p-value threshold of 0.01, giving this association only marginal statistical significance in the main complete dataset. Therefore, this result should be treated with caution as a potential chance finding. This association could be heavily confounded by the detection of anemia or iron deficiency in the mother. We have taken account of the potential confounding of maternal hemoglobin in a sensitivity analysis which did not change this result much. However, the detection of iron deficiency during antenatal care by measuring serum ferritin concentration could still constitute unmeasured confounding.

Our findings suggest that the markers for maternal iron status used in this study (dietary iron intake and hemoglobin concentrations) are unlikely to be related to childhood indicators of cardiovascular health at 10 years. However, they do not exclude a relationship that could become apparent later in the offspring’s lives. In the previous analysis in ALSPAC by Brion et al which reported an association between maternal anemia and offspring BP at 7 years only in women who did not take iron supplements during pregnancy, the sample size with data on maternal hemoglobin (before stratification by supplement intake) was considerably smaller (n = 1255) than our analysis (n = 2958) [14]. Also the definition of early anemia differed in that analysis in that it included both the first and second trimester, while ours used hemoglobin concentrations before 18 weeks gestation. In our analysis, using a bigger sample, stratification by intake of iron supplements did not change the null result of the association with BP (results available from authors on request).

### Strengths & Limitations

Ours is a relatively large study and the first to examine the association of maternal iron intake and maternal hemoglobin in pregnancy with measures of offspring arterial stiffness, endothelial function and adiposity, as well as with BP We have attempted to assess mediation and effect modification based on existing evidence to explain the mechanisms underlying any observed associations. Maternal hemoglobin in early pregnancy was objectively measured as part of routine antenatal care, and extracted for cohort participants from medical records, making bias due to selection and measurement unlikely. Although anemia does not represent a specific or sensitive measure of body iron stores, it is likely to reflect iron deficiency when it is pronounced, particularly in the first trimester [41].

An important limitation of this study is that a biomarker of maternal iron status during pregnancy, such as serum ferritin, or transferrin receptor was not available in this study. Although there are reservations about the use of serum ferritin as a sole measure of iron status as it is an acute inflammatory marker [42], it remains a better indicator compared to self-reported iron intake or hemoglobin concentration [42]. Dietary iron was assessed by self-report, using a food frequency questionnaire, which may provide less accuracy than more detailed methods of dietary assessment such as weighed food diaries. Furthermore, the long term exposure of interest is cardiovascular events, which we are unable to assess in this cohort due to their young age.

It is also known that heme iron is absorbed better than non-heme iron. Although vegetarians often take iron supplements, these may not be as effective as the heme iron that is missing from their diets, and this may have a bearing on the interpretation of our results. However, results based on hemoglobin concentrations in the blood were broadly consistent with those based on dietary intake, suggesting that the source of the iron was not relevant here.

Iron supplement intake was self-reported and the nature of the questions used in this study mean that we do not know the amount of supplementation or whether the iron supplementation was in the form of iron only or as part of a multivitamin preparation. Therefore the association we observed between maternal reported iron supplementation and offspring systolic BP could be attributable to other micronutrient supplements that the mother was taking as well or our null associations may be masked since iron taken as part of a multivitamin preparation may be less readily absorbed compared to when taken on its own [43], [44], [45]. It is difficult to conduct analyses in a British cohort study of iron-only supplements as the exposure. Firstly because, in the UK, these are not recommended routinely in pregnancy, therefore a small proportion of pregnant women would be expected to use them. Secondly, those who take iron-only supplements are likely to be taking the prescribed high-dose as opposed to the low-dose recommended routinely during pregnancy in other countries.

The study sample analysed to test mediation by cord ferritin was considerably smaller. Therefore, when examining the association between maternal iron supplement intake and offspring systolic BP, lack of statistical power may explain the difference in the results between the complete data and multiple imputation models (tables 3 & 4).

In conclusion, this study suggests that maternal anemia during early pregnancy is not an important determinant of future offspring cardiovascular health, using childhood vascular and adiposity indicators at 10 years. We also did not observe any associations between maternal iron intake in pregnancy with offspring’s vascular markers and adiposity except for a modest inverse association between self-reported maternal iron supplement intake during pregnancy and offspring BP.

## References

[1] McArdle HJ, Andersen HS, Jones H, Gambling L (2006) Fetal programming: Causes and consequences as revealed by studies of dietary manipulation in rats-A review. Placenta 27: 56–60. 16533523 10.1016/j.placenta.2006.01.014

[2] WHO (2010) Micronutrient deficiencies. Geneva: World Health Organisation.

[3] Bergmann RL, Gravens-Müller L, Hertwig K, Hinkel J, Andres B, et al (2002) Iron deficiency is prevalent in a sample of pregnant women at delivery in Germany. European Journal of Obstetrics & Gynecology and Reproductive Biology 102: 155–160. 11950483 10.1016/s0301-2115(01)00609-1

[4] Lone F, Qureshi R, Emanuel F (2004) Maternal anaemia and its impact on perinatal outcome. Tropical Medicine & International Health 9: 486–490. 15078267 10.1111/j.1365-3156.2004.01222.x

[5] Allen L (2000) Anemia and iron deficiency: effects on pregnancy outcome. American Journal of Clinical Nutrition 71(suppl): 1280S–1284S. 10799402 10.1093/ajcn/71.5.1280s

[6] Zhou LM, Yang WW, Hua JZ, Deng CQ, Tao X, et al (1998) Relation of Hemoglobin Measured at Different Times in Pregnancy to Preterm Birth and Low Birth Weight in Shanghai, China. American Journal of Epidemiology 148: 998–1006. 9829872 10.1093/oxfordjournals.aje.a009577

[7] Baker PN, Wheeler SJ, Sanders TA, Thomas JE, Hutchinson CJ, et al (2009) A prospective study of micronutrient status in adolescent pregnancy. American Journal of Clinical Nutrition 89: 1114. 19244368 10.3945/ajcn.2008.27097

[8] Scholl TO (2005) Iron status during pregnancy: Setting the stage for mother and infant. American Journal of Clinical Nutrition 81 (suppl): 1218S–1222S. 10.1093/ajcn/81.5.1218 15883455

[9] Gambling L, McArdle HJ (2004) Iron, copper and fetal development. Proceedings of the Nutrition Society 63: 553–562. 15831127 10.1079/pns2004385

[10] Gambling L, Danzeisen R, Fosset C, Andersen H, Dunford S, et al (2003) Iron and copper interactions in development and the effect on pregnancy outcome. J Nutr 133: 1S–3S. 10.1093/jn/133.5.1554S 12730464

[11] Gambling L, Dunford S, Wallace D, Zuur G, Solanky N, et al (2003) Iron deficiency during pregnancy affects postnatal blood pressure in the rat. J Physiol 552: 603–610. 14561840 10.1113/jphysiol.2003.051383 PMC2343386

[12] Crowe C, Dandekar P, Fox M, Dhingra K, Bennet L, et al (1995) The effects of anaemia on heart, placenta and body weight, and blood pressure in fetal and neonatal rats. J Physiol 488: 515–519. 8568690 10.1113/jphysiol.1995.sp020986 PMC1156690

[13] Gambling L, Dunford S, McArdle H (2004) Iron deficiency in the pregnant rat has differential effects on maternal and fetal copper levels. J Nutr Biochem 15: 366–372. 15157943 10.1016/j.jnutbio.2003.12.009

[14] Brion M, Leary S, Davey Smith G, McArdle H, Ness A (2008) Maternal anemia, iron intake in pregnancy, and offspring blood pressure in the Avon Longitudinal Study of Parents and Children. Am J Clin Nutr 88: 1126–1133. 18842803 10.1093/ajcn/88.4.1126

[15] Belfort MB, Rifas-Shiman SL, Rich-Edwards JW, Kleinman KP, Oken E, et al (2008) Maternal iron intake and iron status during pregnancy and child blood pressure at age 3 years. International Journal of Epidemiology 37: 301–308. 18263646 10.1093/ije/dyn002 PMC2650811

[16] Whincup P, Cook D, Papacosta O, Walker M, Perry I (1994) Maternal factors and development of cardiovascular risk: evidence from a study of blood pressure in children. Journal of human hypertension 8: 337–344. 8064780

[17] Bergel E, Haelterman E, Belizan J, Villar J, Carroli G (2000) Perinatal factors associated with blood pressure during childhood. American Journal of Epidemiology 151: 594. 10733041 10.1093/oxfordjournals.aje.a010247

[18] Godfrey KM, Forrester TE, Barker DJ, Jackson AA, Landman Bogues JP, et al (1994) Maternal nutritional status in pregnancy and blood pressure in childhood. Br J Obstet Gynaecol 101: 398–403. 8018610 10.1111/j.1471-0528.1994.tb11911.x

[19] Law CM, Barker DJ, Bull AR, Osmond C (1991) Maternal and fetal influences on blood pressure. British Medical Journal 66: 1291. 10.1136/adc.66.11.1291 PMC1793274 1755640

[20] Agrawal RMD, Tripathi AM, Agarwal KN (1983) Cord blood haemoglobin, iron and ferritin status in maternal anaemia. Acta Pædiatrica 72: 545–548. 10.1111/j.1651-2227.1983.tb09768.x 6624429

[21] Kaneshige E (1981) Serum ferritin as an assessment of iron stores and other hematologic parameters during pregnancy. Obstetrics & Gynecology 57: 238. 7465131

[22] Singla PN, Tyagi M, Shankar R, Dash D, Kumar A (1996) Fetal iron status in maternal anemia. Acta Paediatrica 85: 1327–1330. 8955460 10.1111/j.1651-2227.1996.tb13919.x

[23] Lisle S, Lewis R, Petry C, Ozanne S, Hales C, et al (2003) Effect of maternal iron restriction during pregnancy on renal morphology in the adult rat offspring. British Journal of Nutrition 90: 33–39. 12844373 10.1079/bjn2003881

[24] Gibney MJ, Margetts BM, Kearney JM, Arab L (2004) Public Health Nutrition. Oxford: Blackwell Publishing.

[25] Collings R, Harvey LJ, Hooper L, Hurst R, Brown TJ, et al (2013) The absorption of iron from whole diets: a systematic review. The American Journal of Clinical Nutrition 98: 65–81. 23719560 10.3945/ajcn.112.050609

[26] Alwan NA, Greenwood DC, Simpson NAB, McArdle HJ, Godfrey KM, et al (2011) Dietary iron intake during early pregnancy and birth outcomes in a cohort of British women. Human Reproduction 26: 911–919. 21303776 10.1093/humrep/der005 PMC3057752

[27] Fraser A, Macdonald-Wallis C, Tilling K, Boyd A, Golding J, et al.. (2012) Cohort Profile: The Avon Longitudinal Study of Parents and Children: ALSPAC mothers cohort. International Journal of Epidemiology. 10.1093/ije/dys066 PMC3600619 22507742

[28] Boyd A, Golding J, Macleod J, Lawlor DA, Fraser A, et al (2013) Cohort Profile: The ‘Children of the 90 s’–the index offspring of the Avon Longitudinal Study of Parents and Children. International Journal of Epidemiology 42: 111–127. 22507743 10.1093/ije/dys064 PMC3600618

[29] Rogers I, Emmett P (1998) Diet during pregnancy in a population of pregnant women in South West England. ALSPAC Study Team. Avon Longitudinal Study of Pregnancy and Childhood. European journal of clinical nutrition 52: 246. 9578336 10.1038/sj.ejcn.1600543

[30] Holland B, Welch A, Unwin I, Buss D, Paul A, et al.. (1991) McCance and Widdowson’s the composition of food. Cambridge: The Royal Society of Chemistry.

[31] WHO (2001) Iron deficiency anemia assessment prevention and control: a guide for program managers. Geneva: World Health Organization.

[32] Donald AE, Charakida M, Falaschetti E, Lawlor DA, Halcox JP, et al (2010) Determinants of vascular phenotype in a large childhood population: the Avon Longitudinal Study of Parents and Children (ALSPAC). European Heart Journal 31: 1502. 20421227 10.1093/eurheartj/ehq062 PMC2912638

[33] Halcox JPJ, Donald AE, Ellins E, Witte DR, Shipley MJ, et al (2009) Endothelial function predicts progression of carotid intima-media thickness. Circulation 119: 1005–1012. 19204308 10.1161/CIRCULATIONAHA.108.765701

[34] Halcox JPJ, Schenke WH, Zalos G, Mincemoyer R, Prasad A, et al (2002) Prognostic value of coronary vascular endothelial dysfunction. Circulation 106: 653–658. 12163423 10.1161/01.cir.0000025404.78001.d8

[35] Cruickshank JK, Rezailashkajani M, Goudot G (2009) Arterial Stiffness, Fatness, and Physical Fitness: Ready for Intervention in Childhood and Across the Life Course? Hypertension 53: 602. 19273738 10.1161/HYPERTENSIONAHA.108.128033

[36] Tamura T, Goldenberg RL, Hou J, Johnston KE, Cliver SP, et al (2002) Cord serum ferritin concentrations and mental and psychomotor development of children at five years of age. Obstetrical & Gynecological Survey 57: 493. 10.1067/mpd.2002.120688 11865266

[37] Brion MJA, Ness AR, Rogers I, Emmett P, Cribb V, et al (2010) Maternal macronutrient and energy intakes in pregnancy and offspring intake at 10 y: exploring parental comparisons and prenatal effects. American Journal of Clinical Nutrition 91: 748. 20053880 10.3945/ajcn.2009.28623 PMC2822901

[38] Boyd A, Golding J, Macleod J, Lawlor DA, Fraser A, et al.. (2012) Cohort Profile: The ‘Children of the 90s’–the index offspring of the Avon Longitudinal Study of Parents and Children. International Journal of Epidemiology. 10.1093/ije/dys064 PMC3600618 22507743

[39] FSA (2003) National Diet and Nutrition Survey: Adults aged 19 to 64, Volume 3 London: Food Standards Agency.

[40] Royston P (2004) Multiple imputation of missing values. Stata Journal 4: 227–241.

[41] Milman N (2006) Iron and pregnancy–a delicate balance. Annals of Hematology 85: 559–565. 16691399 10.1007/s00277-006-0108-2

[42] Zimmermann M (2008) Methods to assess iron and iodine status. British Journal of Nutrition 99: S2–S9. 10.1017/S000711450800679X 18598585

[43] Gambling L, Andersen H, McArdle H (2008) Iron and copper, and their interactions during development. Biochemical Society Transactions 36: 1258–1261. 19021536 10.1042/BST0361258

[44] Gambling L, Danzeisen R, Fosset C, Andersen HS, Dunford S, et al (2003) Iron and copper interactions in development and the effect on pregnancy outcome. The Journal of Nutrition 133: 1554S–1556S. 12730464 10.1093/jn/133.5.1554S

[45] Kelleher SL, Lönnerdal B (2006) Zinc supplementation reduces iron absorption through age-dependent changes in small intestine iron transporter expression in suckling rat pups. The Journal of Nutrition 136: 1185–1191. 16614402 10.1093/jn/136.5.1185

